# Cellular and Molecular Mediators of Neuroinflammation in the Pathogenesis of Parkinson's Disease

**DOI:** 10.1155/2013/952375

**Published:** 2013-06-27

**Authors:** Sandeep Vasant More, Hemant Kumar, In Su Kim, Soo-Yeol Song, Dong-Kug Choi

**Affiliations:** Department of Biotechnology, College of Biomedical and Health Science, Konkuk University, Chungju 380-701, Republic of Korea

## Abstract

Neuroinflammation is a host-defense mechanism associated with restoration of normal structure and function of the brain and neutralization of an insult. Increasing neuropathological and biochemical evidence from the brains of individuals with Parkinson's disease (PD) provides strong evidence for activation of neuroinflammatory pathways. Microglia, the resident innate immune cells, may play a major role in the inflammatory process of the diseased brain of patients with PD. Although microglia forms the first line of defense for the neural parenchyma, uncontrolled activation of microglia may directly affect neurons by releasing various molecular mediators such as inflammatory cytokines (tumor necrosis factor-**α**, interleukin [IL]-6, and IL-1**β**), nitric oxide, prostaglandin E2, and reactive oxygen and nitrogen species. Moreover, recent studies have reported that activated microglia phagocytose not only damaged cell debris but also intact neighboring cells. This phenomenon further supports their active participation in self-enduring neuronal damage cycles. As the relationship between PD and neuroinflammation is being studied, there is a realization that both cellular and molecular mediators are most likely assisting pathological processes leading to disease progression. Here, we discuss mediators of neuroinflammation, which are known activators released from damaged parenchyma of the brain and result in neuronal degeneration in patients with PD.

## 1. Introduction

Parkinson's disease (PD) is a frequent neurological disorder of the basal ganglia characterized by the progressive loss of dopaminergic neurons, mainly in the substantia nigra pars compacta (SNpc) [1], cytoplasmic inclusions of aggregated proteins, and neuroinflammation [2, 3]. Several hypotheses have been postulated regarding the possible causes for neuronal degeneration in patients with PD. These include genetic factors, environmental toxins, mitochondrial dysfunction, and free radical-mediated cell death [4–6]. Although there is less evidence suggesting that neuroinflammation is the primary trigger causing neurodegeneration, preclinical and epidemiological data now strongly suggest that chronic neuroinflammation may be a slow and steady reason for neuronal dysfunction during the asymptomatic stage of PD [7]. Neuroinflammation induced by exposure to either infectious agents or toxicants with proinflammatory characteristics is now increasingly recognized as a major contributor to the pathogenesis of PD [8]. Whitton in 1988 initially suggested the involvement of inflammation in PD by describing upregulation of major histocompatibility complex (MHC) molecules in patients with PD [9]. The hallmarks of neuroinflammation are the presence of activated microglia and reactive astrocytes in the parenchyma (neurons, astrocytes, and endothelial cells) of the central nervous system (CNS), direct participation of the adaptive immune system, increased production of cytokines, chemokines, prostaglandins, a cascade of complement proteins, and reactive oxygen and nitrogen species (ROS/RNS), which in some cases can result in disruption of the blood-brain barrier (BBB) [10]. The extent to which neuroinflammation and peripheral immune responses contribute towards the development of PD or modify its course is not exactly known. In fact, dysregulation of the neuroimmune system has been postulated by many to be one of the underlying causes of the chronic nature of PD.

Several lines of evidence support the hypothesis that glial reactive and inflammatory processes participate in the cascade of events leading to neuronal degeneration [11]. One of the earlier studies reporting neuroinflammation in PD involved a quantitative confirmation of the astroglial reaction using glial fibrillary acidic protein (GFAP) immunostaining in the substantia nigra (SN) of patients with PD [12]. Fundamental work by McGeer et al. [13], over two decades ago, first identified significantly increased levels of human leucocyte antigen-DR-positive microglia in the postmortem brains of patients with PD [14]. Following these reports, an increased number of activated microglial cells had consistently been reported in the neuroinflammatory pathogenesis of PD [11]. Initially, the pathological role played by these glial cells was not fully understood, but activated microglial cells have a deleterious effect on dopaminergic neurons. Microglial cells represent resident brain macrophages that are transformed into activated immunocompetent antigen-presenting cells during the pathological process [15]. Microglia in PD have been observed to grow densely in the striatum and SN with increased expression of proinflammatory mediators, including tumor necrosis factor alpha (TNF-*α*), interleukin-1*β* (IL-1*β*) [16], IL-2, IL-4, IL-6, transforming growth factor-*α* (TGF-*α*), cyclooxygenase-2 (COX-2), and inducible nitric oxide synthase (iNOS) [14]. TGF-*β*1 and *β*2 have also been detected by several investigators in the cerebrospinal fluid (CSF) and brain parenchyma of patients suffering from PD [17]. Taken together, these data indicate that glial cells are one of the most important cells involved in mediating neuroinflammatory events in PD [18].

In addition to glial cells, other cells may also participate in the neuroinflammatory processes in PD. Increasing evidence now demonstrates the involvement of both innate and adaptive immune responses in the pathophysiology of PD [19–21]. Innate immunity does not require the presence of a specific antigen to elicit an immune response, whereas adaptive immunity is activated when specific antigens are presented and recognized by lymphocytes. In contrast, endogenous pathological antigens that normally do not occur in physiological conditions may also initiate adaptive immune responses [22]. Indeed, Hirsch et al., in their various experiments, reported a small number of CD8-positive T lymphocytes in the vicinity of degenerating neurons in the SN of a patient with PD [18]. In line with this observation, an increased density of interferon-(IFN-) *γ*-positive cells with lymphocytes in the SN of patients with PD has also been reported [18]. Taken together, these data indicate that injured dopaminergic neurons release immunogenic mediators which have the potential to provoke detrimental innate and adaptive immune responses thereby amplifying the neuroinflammatory process in PD. Furthermore, mounting evidence suggests that BBB permeability may be modulated under neuroinflammatory conditions, and trafficking of leukocytes and peripheral macrophages into the brain becomes a normal process that must be tightly regulated to promote brain homeostasis and avoid the neuronal demise [10, 23]. These pathomechanisms not only produce complex cross-talk between the peripheral immune system and CNS but also highlight the interactions between microglial cells and other brain parenchymal cells [18]. Therefore, identifying and understanding the nature and role of the neuroinflammatory mediators involved in the pathogenesis of PD might provide us with various options to target these neuroinflammatory pathways to help curb neuronal death in PD. This review describes various cellular and molecular mediators of neuroinflammation which occur in response to or as part of the ongoing disease progression in PD.

## 2. Mediators of Neuroinflammation

### 2.1. Role of Microglia as a Mediator of Neuroinflammation in PD

Glia are composed of three distinct cells types named as microglia, astrocytes, and oligodendrocytes [24]. Several populations of macrophages are present in different compartments of healthy brain tissue, each with a distinct phenotype and morphology. The most abundant of these macrophages are the microglia, the resident macrophages of the brain parenchyma [25]. Microglia maintains homeostasis and performs immune surveillance by continuously examining their environment by extending cellular protrusions [26]. With a plentitude of ion channels, cytokines, Toll-like receptors, and chemokine receptors [21], microglia promptly reacts in response to subtle alterations in their microenvironment such as alterations in ion homeostasis and brain insults, ranging from aggregated proteins to pathogens [27]. Microglial cells are generally quiescent in the normal brain, with their cell bodies barely visible and few detectable fine ramified processes. However, resting microglial cells quickly proliferate, become hypertrophic, and persistently increase expression of a large number of marker molecules such as CD11b, CD68, and MHC-I and II molecules [28] and are further transformed to macrophage-like cells in patients with PD [11, 29, 30]. Microglia may be transformed into M1 or M2 macrophages depending upon the type of stimulus [31, 32]. It is now apparent that microglia occur in many different phenotypes that cannot be readily divided into a small number of discrete subsets following tissue injury [33]. The SN is relatively rich in microglia compared with other brain regions [34, 35]. In addition, a reduced level of intracellular glutathione in the SN dopaminergic neurons makes them much more susceptible to a variety of insults, including activated microglial-mediated injury and oxidative stress [35]. This observation indicates that localization of microglia in the SN predisposes dopaminergic neurons to immunological insult in patients with PD [36].

The selective acute degeneration of dopaminergic cells in the SN can be induced by toxins such as 6-hydroxydopamine (6-OHDA), 1-methyl-4-phenyl-1,2,3,6-tetrahydropyridine (MPTP), and rotenone. In all of these rodent toxin models, dopaminergic cells of the SN degenerate over a period of a few days. A recent study comparing the commonly used medial forebrain or intrastriatal injection of 6-OHDA showed rapid degeneration induced by the toxin, which was accompanied by activation of microglia as assessed by the upregulation of the complement type 3 receptor [37]. Similarly, MPTP-induced neurodegeneration is associated with activated microglia [38, 39]. It has become increasingly apparent that there are various triggers through which microglia are activated to elicit their neurotoxic response. Interestingly, while these diverse toxins exhibit several mechanisms of microglial activation, toxin-induced activation of NADPH oxidase is the most common pathway through which microglia exerts neurotoxicity [36]. Apart from this toxin-induced microglial activation, various other triggers involved in microglial activation include immunological insults such as IFN-*γ*, lipopolysaccharide (LPS), chemokines (CCL5, CCL2, and CXCL10), neurotransmitters, gangliosides, the CD40 ligand, proteases such as thrombin [40], tissue plasminogen activator [41], matrix metalloproteinase-3 (MMP-3) [21], endogenous disease proteins, and neuronal injury itself [42]. Among these activators, LPS-induced neuroinflammation is one of the most accepted and widely used endotoxin models that induces a strong neuroinflammatory response in BV-2 microglial cells [43, 44] or when injected directly into the vicinity of the SN [45]. Recent findings demonstrate that neurons are not simply passive targets of microglia but rather control microglial activation [3, 46, 47]. A variety of signals that neurons use to modulate microglia can be categorized into excitatory and inhibitory signals. Inhibitory signals from neurons constitutively maintain microglia in their quiescent state and antagonize proinflammatory activity, whereas excitatory signals are inducible and incite activation of microglia under pathological conditions towards a beneficial or detrimental phenotype. Thus, various neuronal signaling molecules actively modulate microglial functions and contribute to the inflammatory milieu of PD [46]. The two subclasses of neuronal inhibitory signals are called released and membrane-bound signals. Released inhibitory signals can be CX3CL1, CD22, TGF-*β*, brain derived neurotrophic factor (BDNF), neurotrophin-3, nerve growth factor, or neurotransmitters. The release site for neuronal inhibitory signals is not yet known but is thought to be related to synaptic activity [48, 49]. Membrane-bound neuronal inhibitory signals consist of several molecules from the immunoglobulin superfamily including CD200 [50], CD22, and CD47, which are expressed or secreted by neurons that bind to receptors on microglia [48, 51, 52]. These immunoreceptors, so-called tyrosine-based inhibitory motif receptors, contain a cytoplasmic motif that inhibits activation of microglia. This mechanism of maintaining microglia in their silent state also depends upon the density of the ligands expressed by neurons, which are reduced during PD and will thus shift the level of inhibitory tone to activation in microglia [25]. In parallel with the inhibitory signals, neuronal excitatory signals are classified into released and membrane-bound signals. The released excitatory signals control various aspects of microglia function and can be listed as chemokines (CX3CL1, CCL21, and CXCL10) [53–55], glutamate [56], purines (ATP and UTP) [57, 58], or MMP-3 [59, 60] which modulate various aspects of microglia function. Taken together, microglia are maintained in a quiescent state under normal physiological conditions by the orchestrated action of neurons and astrocytes; however, microglia are rapidly activated when integrity of a neuron is disrupted in PD, probably as a result of both direct activation signals from neurons or loss of inhibitory signals by neurons [47].

While it is clear that microglia becomes activated upon neuronal damage, proteases known to modify the extracellular matrix (ECM) may also be a critical mechanism through which damaged neurons activate microglia to produce extracellular superoxide. Earlier reports from Chang et al. emphasized the critical role of ECM proteins in the interactions between microglia and neurons [61]. Later, it was found that MMP-3, a proteinase that degrades ECM components, is released following damage to dopaminergic neurons exposed to 1-methyl-4-phenylpyridinium (MPP^+^), exerting neurotoxicity to the dopaminergic neurons [59]. It was also observed that exposure of mesencephalic neuron/glia cultures to MPP^+^ results in a dose-dependent increase in MMP-3 protein expression, both in conditioned media and in cell lysates, indicating that death of dopaminergic neurons upregulates MMP-3 expression. These data suggest that MMP-3 is a crucial mediator released upon damage to dopaminergic neurons and that it activates microglia to further propagate neuronal cell death in PD [42]. Besides MMP-3, damaged or dying dopaminergic neurons release neuromelanin to activate microglia in the SN of patients with PD [62, 63]. Neuromelanin has the potential to be neurotoxic, because excess neuromelanin inhibits the function of dopaminergic neurons, proteasomes and induces the production of toxic factors such as TNF-*α*, IL-6, and nitric oxide (NO) [64, 65]. Among the proinflammatory mediators released during microglial activation, some act synergistically to produce inflammation-related neuronal damage. Hence, the identification of several potential mediators of microglia activation has allowed a general classification of how microglia respond to stimuli. Microglia can also be activated by products of the classical complement cascade and by chromogranin A [66, 67], which has been reported to occur in the PD SN [68]. Among the array of mediators released, superoxide is necessary for both the induction and amplification of neurotoxicity in PD [69, 70]. NADPH oxidase (PHOX) is the major superoxide-producing enzyme in microglia [71]. Activation of PHOX results in translocation of its cytosolic subunits to the cellular membrane to form a functional enzyme that not only generates superoxide but also controls the levels of other proinflammatory neurotoxic mediators produced by microglia in PD [72]. It has been revealed that PHOX is closely paired with Mac-1 and plays an important role in microglia-mediated neuroinflammation and neurotoxicity [73]. Therefore, coupling between PHOX and Mac-1 might be a central mechanism responsible for the oxidative damage induced by reactive microgliosis that results in progressive neuroinflammation in PD [36].

**Figure 1:**
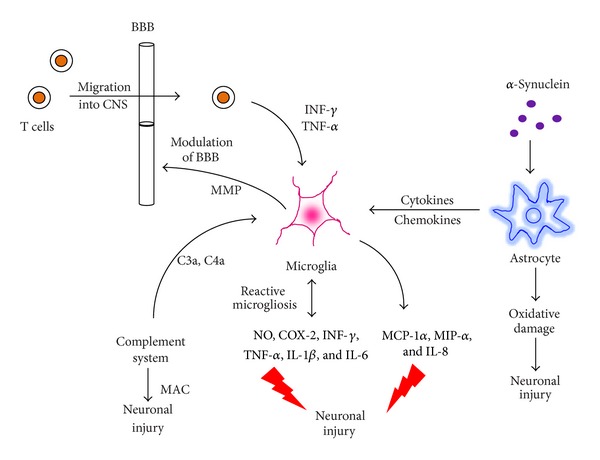
Microglial and astroglial cells become activated during the pathogenesis of Parkinson's disease under the influence of various proinflammatory triggers, including *α*-synuclein, the complement system, and cytokines released from infiltrated T cells. Activated microglial and astroglial cells further release various neuroinflammatory mediators, including NO, COX-2, IFN-*γ*, TNF-*α*, IL-1*β* & IL-6, chemokines including MCP-1*α*, MIP-*α* and CXCL-8, and MAC which have deleterious effect on neuronal survival. Abbreviations: NO: nitric oxide, COX-2: cyclooxygenase, INF-*γ*: interferon-*γ*, TNF-*α*: tumor necrosis factor-*α*, IL-1*β*: interleukin-1*β*, IL-6: interleukin-6, MCP-1*α*: monocyte chemotactic protein-1, MIP-*α*: microphage inflammatory protein, IL-8: interleukin-8, MAC: membrane attack complex, *α*-syn: *α*-synuclein, MMP: matrix metalloprotein, BBB: blood brain barrier, C3a: complement component 3a, and C4a: complement component 4a.

To date, one of the best elucidated cytotoxic mechanisms induced by proinflammatory cytokines in PD is activation of iNOS. iNOS mediates the synthesis of high levels of NO, which is toxic to dopaminergic neurons [74]. The density of glial cells expressing iNOS increases significantly in the SN of patients with PD compared with that in control subjects [75]. The induction of NOS produces high levels of NO and superoxide radicals for a prolonged period of time. These two ROS can either directly or indirectly promote neuronal death in PD [76]. Prostaglandins and their synthesizing enzymes, such as COX-2, represent a second group of potential culprits in PD. Expression of COX-2 along with the levels of its product, prostaglandin E2 (PGE_2_), increases significantly in glial cells of the SNpc, which are responsible for many of the cytotoxic effects to dopaminergic neurons in PD [77]. Many reports have demonstrated increased expression of COX-2 in PD [77, 78]. In fact, several studies have observed upregulation of COX-2 in animal models of PD [79–81]. Increased COX-2 expression has also been shown in the SN of postmortem PD specimens compared to that in normal controls [75, 82]. Inhibiting COX-2 [80, 81, 83, 84] and transgenic mice lacking COX-2 expression [85] in models of PD has been demonstrated to increase survival of dopaminergic neurons. Release of *α*-synuclein (*α*-syn) from neuronal damage could also incite the production of proinflammatory mediators such as PGE_2_ from microglia [86], thus, contributing to the progression of nigral neurodegeneration. It has recently been observed that modifying ubiquitin carboxy-terminal hydrolase L1 by cyclopentenone prostaglandins causes protein unfolding and aggregation. Hence, the deleterious effect of COX-2 in PD could be due to the production of cyclopentenone prostaglandins [87]. Although the exact causal link between neuronal injury and microglial activation in PD remains controversial, one of the earliest reported harmful effects demonstrated to cause demise of dopaminergic neurons was microglial-mediated release of proinflammatory cytokines, including IFN-*γ* [20] IL-1*β*, TNF-*α*, IL-2, and IL-6 [88] with elevated levels of TNF-*α* receptor R1 (p55), bcl-2, soluble Fas, caspase-1 and caspase-3 in postmortem striatum, SN, and CSF of patients with PD [89, 90]. These cytokines, in turn, propagate and intensify neuroinflammation and cause irreversible destruction of SN dopaminergic neurons [91] by a number of mechanisms, including upregulating isoforms of phospholipases A2, generating platelet-activating factor, stimulating NOS, and activating calpain [9, 92]. We have briefly summarized some of the major mediators of neuroinflammation during the pathogenesis of PD in Figure 1.

### 2.2. Role of T Cells as a Mediator of Neuroinflammation in PD

The brain has long been considered an immune privileged system, as it is protected by the BBB. However, recent findings demonstrate that both the innate and adaptive immune systems play a very critical role disrupting BBB permeability and mediating the pathogenesis of PD via their ability to supply the required signals for antigen presentation and to act as final effectors by T cells [25, 93, 94]. For example, infiltration of T cells has been found in the brains of patients with PD [95] as well as significant infiltration of adoptively transferred immune splenocytes into the brains of MPTP-intoxicated mice and localization within the inflamed SN [93]. Similarly, a recent study of MPTP model demonstrated the necessity of T cells to mediate degeneration of dopaminergic neurons and that dopaminergic neuronal loss is exacerbated by T cells [96]. Increased mutual coexpression of CD4 and CD8 by CD45R0^+^ T cells with increased expression of CD25, TNF-*α* receptors, and diminished expression of IFN-*γ* receptors suggests that subsets of T cell are indeed activated in patients with PD [97]. The influence of infiltrating T cells on dopaminergic neurons has been demonstrated in mice lacking T lymphocytes, wherein death of dopaminergic neurons was significantly attenuated in both types of mice as compared to that in wild-type animals [22]. It was also observed that a subset of CD4^+^ T cells, rather than CD8^+^ T cells, mediate the cytotoxic effects on dopaminergic neurons, as survival of dopaminergic neurons after MPTP administration increases in CD4-deficient mice but not in CD8-deficient mice [22]. Further analysis showed that CD4^+^ T cells exert their cytotoxicity through the Fas-FasL pathway rather than through IFN-*γ* secretion [98]. Early evidence from the SN by autopsy of patients with PD showed increased numbers of CD8^+^ T cells in close proximity with activated microglia and degenerating neurons [99]. More recently, both CD4^+^ and CD8^+^ T cells have been discovered within the SN of patients with PD [22]. Significant level of unrepaired single-strand DNA breaks and a number of micronuclei are more also observed in lymphocytes and activated T cells from patients with PD due to inflammation and exposure to ROS than those in age-matched controls [100, 101]. CD8^+^ T cells were the first type of peripheral T lymphocytes to be located in the postmortem brain from a PD patient [13]. Later, infiltration of CD4^+^ and CD8^+^ T cells was found in the SN and striatum of MPTP-intoxicated mice [93, 102]. A more recent report provided substantial evidence of significant infiltration of CD4^+^ and CD8^+^ T cells in the SN of patients with PD and in MPTP-intoxicated mice [22]. Increased frequencies of activated CD4^+^ T cells expressing Fas [103], increased IFN-*γ*-producing Th1 cells, decreased IL-4-producing Th2 cells, and a decrease in CD4^+^, CD25^+^ T cells have been found in the peripheral blood of patients with PD [104]. These data suggest complex roles for CD4^+^ subsets of T cells in mediating the development of PD.

Compelling evidence suggests the possible involvement of the BBB, including changes in lymphocytic subpopulations in the blood and CSF of patients with PD [91, 103]. Moreover, an increased proportion of *γδ*-positive lymphocytes, thought to play a role in infections and autoimmunity, have also been reported in the CSF and blood of patients with PD [105]. These data suggest that infiltration of immune cells across the BBB into the brain participates in the pathophysiology of PD. This infiltration of peripheral lymphocytes into the brain through the BBB occurs mainly because activated microglia and monocytes in the brains of patients with PD release proinflammatory cytokines and chemokines that act on the vascular endothelium to induce upregulation of cell adhesion molecules, including vascular cell adhesion molecule-1 and intercellular adhesion molecule-1 (ICAM-1) [106] that disrupt the BBB and attract lymphocytes [97] expressing counterreceptors such as leukocyte function antigen-1 (LFA-1) to the neuronal injury site [106]. Activated T cells further release proinflammatory cytokines such as IFN-*γ* and TNF-*α*, which positively induce the expression of costimulatory molecules and MHC-II on microglial cells [107]. When these activated microglial molecules bind to their respective receptors on T cells, they are transformed into effector T cells. Among the activated T cells, CD8^+^ T lymphocytes mediate direct cytotoxic lysis of target cells, recruit and activate accessory cells via proinflammatory mediators, whereas activated CD4^+^ T lymphocytes induce B cells to produce high-affinity antibodies [91]. Nitrated-*α*-syn (N-*α*-syn), a misfolded protein present in intraneuronal inclusions or Lewy bodies in patients with PD, can be released to the extraneuronal space and cross the BBB to enter the cervical lymph nodes, where it can activate antigen-presenting cells [93]. This phenomenon has also been observed in MPTP-intoxicated mice, wherein *α*-syn drains to cervical lymph nodes [93]. As a novel epitope, N-*α*-syn can be processed and presented to naive T cells, thus, stimulating them to expand into different subsets of effector T cells. Peripherally activated effector T cells, such as Th1 or Th17 cells, can also cross the BBB and reach inflammatory sites within the brain where they activate microglia and release proinflammatory cytokine IL-17 and secrete cytolytic enzymes such as granzyme B [108]. Although a dysfunctional BBB in patients with PD may show some leakiness [109], it may not be sufficient to allow unrestricted lymphocyte infiltration because CD4/CD8 ratios are 1 : 4.8 [22] compared with the typical 2 : 1 ratio expected for peripheral T cells performing surveillance functions. Thus, the mechanisms by which these T cells gain access to the SN, their activation state, and their functions are questions that remain to be answered [97].

In summary, infiltrating subsets of T cells may induce excessive microglial-mediated inflammation and oxidative stress that exacerbate neuroinflammation in PD. Further study is needed to identify the exact roles of specific subsets of CD4^+^ T cells on the pathogenic progression of PD [98]. Studies suggest that the adaptive immune system similar to the innate immune system not only responds to but also actively participates in the pathogenesis of PD. However, more work needs to be done to determine if and how they could serve as a potential target for PD therapy [36].

### 2.3. Role of Astrocytes as a Mediator of Neuroinflammation in PD

The majority of findings from research on PD point toward microglia as the major mediator of neuroinflammation, but the astrocytic reaction is another well-known neuropathological characteristic in the SN of patients with PD [110]. Astrocytes function as supportive cells for neurons and maintain homeostasis and other neuronal functions [111]. Compared to microglia, their role as innate immune cells is somehow less appreciated. Nonetheless, astrocytes form the glia limitans around blood vessels, preventing entry of immune cells via the BBB into the CNS parenchyma [112]. Emerging evidence has focused upon the importance of astrocytes in the regulation of neuroinflammation in PD [113, 114]. The SNpc of many postmortem PD cases had been observed to have an increased number of astrocytes and GFAP immunoreactivity [115] with a decrease in glial-derived neurotrophic factor, BDNF, and ciliary neurotrophic factor [116]. The amount of GFAP-positive astrocytes is inversely proportional to the demise of dopaminergic neurons [12], indicating that dopaminergic neurons are more susceptible to the degenerative process wherein there are fewer astrocytes. Activation of astrocytes is characterized by the formation of hypertrophic and glial scars, which hinder axonal regeneration [42], enlarged cell bodies, and projections into the injured area [117] that seem to be mediated by proteoglycans [118]. During inflammatory conditions, astrocyte-derived granulocyte macrophage-colony stimulating factor, IL-6, CCL2, and CCL5 regulates migration, activation, and proliferation of microglia [119]. Astrocytes may detect neuron-derived *α*-sys as a degenerative marker released by neurons and get activated to protect neurons. However, such reactive astrocytes are exposed to increasing toxicity from *α*-sys oligomers and/or protofibrils, until they no longer serve a protective function [120].

Astrocytes play a major role mediating MPTP toxicity, as the active metabolite of MPTP, MPP^+^ is extruded into the extracellular space from astrocytes and further enters into dopaminergic neurons and induces neurotoxicity by inhibiting complex I in the mitochondrial electron transport chain [113]. Astrocytic activation parallels the development of dopaminergic cell death in the SNpc and striatum, whereas GFAP expression remains high even after most dopaminergic neurons have died due to administration of MPTP. These findings suggest that the astrocytic reaction occurs after neuronal cell death in PD [121]. In a recent study, it was demonstrated that *α*-syn released from neuronal cells can also be transferred to and accumulate in astrocytes and induce expression of genes associated with immune functions [122]. Proinflammatory cytokines that are differentially expressed in astrocytes in response to extracellular *α*-syn include IL-1*α*, IL-1*β*, IL-6, IL-18, and colony-stimulating factors-1, 2, and 3, suggesting a strong inflammatory response from astrocytes upon exposure to neuron-derived *α*-syn [122]. Exposure to neuron-derived *α*-syn also causes dramatic changes in chemokine expression in astrocytes, including CC-type (CCL-3, 4, 5, 12, 20), CXC-type (CXCL-1, 2, 5, 10, 11, 12, 16), and CX3C-type (CX3CL1) chemokines. These chemokines are involved in a variety of functions, such as recruitment of monocytes and macrophages, migration of microglia and neural progenitors, regulation of microglial activity, proliferation and survival of astrocytes, and synaptic plasticity and transmission [123]. These released chemokines, including CXCL12 and CCL5, induce glutamate release and restart the synthesis of cytokines and chemokines in astrocytes, suggesting their role in glia-glia and glia-neuron communication [122].

Other than cytokines, inflammatory oxidants have emerged as key contributors to PD and MPTP-related neurodegeneration. In this context, myeloperoxidase (MPO), a key oxidant-producing enzyme, which is mostly expressed by reactive astrocytes during inflammation, is upregulated in the ventral midbrain of human patients with PD and in MPTP mice [110]. MPO oxidizes nonreactive nitrite whose concentration is increased in parkinsonism [124] to reactive nitrite (NO_2_^−^) and, thus, nitrosylates many proteins [125]. Reactive nitrites also contribute towards the production of the nonradical oxidant, hypochlorous acid (HOCl), which can damage macromolecules indirectly by fuelling hydroxyl freeradicals or directly by converting amines into chloramines, phenols, and unsaturated bond chlorination [126]. Furthermore, MPO also directly produces HOCl from hydrogen peroxide and chloride anion. Thus, HOCl might directly inflict oxidative damage on dopaminergic neurons [110]. Apart from the direct release of proinflammatory cytokines, including TNF-*α* and IL-6, astrocytes can also be activated by cytokines such as IL-1*β* and TNF-*α* released from microglia, thus, producing ROS and RNS [127]. In support of this observation, a recent study has reported that microglial inflammatory responses are enhanced by astrocytes through a nuclear factor-*κ*B-dependent mechanism leading to increased dopaminergic toxicity [127]. Astrocytes also produce mediators which play a vital role mediating the inflammatory reaction that occurs in the SN of patients with PD. For example, ICAM-1-positive astrocytes are seen in the SN in patients with PD and attract reactive microglia to the area because such microglia carries the LFA-1 counterreceptor [95]. In addition, *α*-syn activates microglia and astrocytes to produce IL-6 and ICAM-1 [128]. This combination further attracts reactive microglia to the site of neuronal injury. The action of *α*-syn on astrocytes is thought to be through receptors, but the identity of these receptors is currently unknown [3].

Activated CD4^+^ T cells express and release several inflammatory factors such as the Fas ligand, a cell-surface molecule in the TNF-*α* family. Fas expression increases in patients with PD and in mice exposed to MPTP [129, 130]. This Fas ligand binds with the Fas receptor expressed on astrocytes and causes a release of various cytokines such as IL-6 and IL-8 and chemokines such as monocyte chemoattractant protein-1 [110]. The detrimental consequence of activating the Fas-Fas ligand pathway in PD has recently been established by several investigators. It has also been reported that mice deficient in Fas are more resistant to MPTP exposure than wild-type controls [131]. The current literature certainly suggests that astrocytes have the ability to modulate the function and survival of dopaminergic neurons in PD.

### 2.4. Role of the Complement System as a Mediator of Neuroinflammation in PD

The complement system is believed to amplify the effectiveness of both the specific and nonspecific immunological defense system. The complement system destroys invading pathogens, encourages inflammation, and supports phagocytosis of waste materials [132]. The complement system has the full ability to recognize molecular patterns associated with injured tissues and dying cells or molecular patterns on pathogens [133]. Various complement proteins, mostly present in tissue fluids and blood, are in the form of soluble monomers. The complement system can be activated by molecules other than antibodies. One such molecule, which is elevated in the SN of patients with PD, is C-reactive protein [134]. Similar evidence about the involvement with the complement system in PD has also been reported wherein membrane attack complex (MAC) together with all complement protein components has been identified on oligodendroglia of the SN and intracellularly in Lewy bodies of patients with sporadic PD [135, 136] and familial PD [137]. Additionally, elevated levels of MAC [135], C-reactive protein, and complement 3 have been observed in the SN and CSF of patients with PD [138, 139]. Increased mRNA levels of complement components have also been found in affected brain regions of PD models [138]. Activation of the complement system leads to a cascade of events ultimately leading to the destruction of cell surface with three different recognized pathways, which share a common juncture at the level of the C3 protein [140, 141].

Products of the activated complement cascade include opsonizing components (C3b, iC3b, and C4b) [142], which stain material for phagocytosis, MAC, and anaphylatoxins (C3a, C4a, and C5a) [142]. The opsonins perform a clearance function, whereas anaphylatoxins are involved in generation of the neuroinflammatory response [143]. In contrast, MAC induces cell death by entering cell membranes and causing organelles to leak. Although MAC destroys foreign cells and viruses, nearby host cells are at a significant risk of lysis if they are not protected by MAC [135, 140]. The complement system also contributes to the secretion of inflammatory cytokines from activated microglial cells [144]. Very recent evidence has demonstrated involvement of the complement system in the pathogenesis of PD, wherein the only cells in the SN and other brain areas that express C1q are microglia [145]. One of the important features of PD is that degeneration of dopaminergic neurons is accompanied by the deposition of extracellular neuromelanin. Degenerating neurons along with neuromelanin granules are opsonized by C1q and phagocytosed by C1q-positive microglia and macrophages in the perivascular spaces and parenchyma. Furthermore, the luminal surfaces of blood vessels in the SN of patients with PD have attached neuromelanin-laden C1q-positive cells. Thus, microglia are capable of clearing cellular debris from degenerating neurons of the SN and phagocytosing cells through the C1q-mediated pathway in PD [145]. Pentraxin is one of the mediators which activates the complement system by binding to the collagen tail of C1q. Pentraxin is an acute-phase protein that is involved in innate immunity and inflammatory response. Glial cells may be the major cellular source of this protein in the CNS. Under the inflammatory milieu of PD, pentraxin proteins secreted by reactive glial cells are detected in the plasma and CSF of patients with PD [146, 147]. Hence, pentraxin could serve as an inflammatory biomarker for PD. Overall, it seems clear that there is a role for the complement system in inflammation-mediated neurodegeneration in PD [138, 140]; hence, research aiming at developing effective inhibitors targeting these sites appears to be worthwhile.

## 3. Conclusion

PD is one of the most common neurodegenerative diseases with a well-established group of symptoms. Although a number of different mechanisms have been considered responsible for the development of PD, none are absolute. Growing evidence from patients and experimental models of PD has indicated that neuroinflammation is one of the driving forces in the pathogenesis of PD. The CNS had been thought to be an immunologically protected organ, but this notion has now undergone considerable reassessment. It has become apparent from a number of reports that various neuronal injury signals from different neuronal cell types in response to environmental insults, involving many mediators, incite and disseminate the ongoing neuroinflammation in PD. We have summarized the evidence wherein neuroinflammatory mediators play a key role in the pathogenesis of PD. Neuroinflammatory mediators have a profound action on CNS cells that differently affect the progress of inflammation and neuronal death. Therefore, regulating the production of neuroinflammatory mediators or their action on respective receptors would be an effective approach to mitigate the inflammatory processes in PD. Thus, further studies are required to form a more comprehensive idea about the role of these neuroinflammatory mediators in PD. Furthermore, it is of significant interest for ongoing research to identify and target various neuroinflammatory mediators released in response to various toxins to help explain how neuronal damage can signal inflammation and propagate neuronal cell death. This knowledge might serve to develop pharmacological strategies for treating the neuroinflammation in PD.
